# Supplementary material for: Complexome profiling of the Chlamydomonas psb28 mutant reveals TEF5 as an early PSII assembly factor
Source: Plant Cell. 2025 Mar 17;37(6):koaf055. doi: 10.1093/plcell/koaf055 (PMC12164590; doi:10.1093/plcell/koaf055)
Supplement: koaf055_Supplementary_Data [file koaf055_supplementary_data.zip › psb28_interactive.zip!/plots/intensity/qualityPlots/peptides/PSAK_Cre17.g724300.t1.2.html]

### Cre17.g724300.t1.2

Trivial name: PSAK  
SeqCoverage [median]: 40.70  
Mass: 11.20 kDa  
  
 

| Sequence | MissedCleavage | Mass [Da] | wtIntensity | muIntensity | Score |
| --- | --- | --- | --- | --- | --- |
| ADGFIGSSTNLIMVASTTATLAAAR | 0 | 2438.25 | 2838.70 | 0.00 | 93.77 |
| FGLAPTVK | 0 | 831.49 | 947594.59 | 619195.62 | 180.70 |
| FGLAPTVKK | 1 | 959.58 | 102106.91 | 112978.24 | 155.88 |
| KNTTAGLK | 1 | 831.48 | 845.16 | 2026.66 | 97.80 |
| NTTAGLK | 0 | 703.39 | 168684.95 | 185431.05 | 138.55 |
| NTTAGLKLVDSK | 1 | 1245.69 | 3172.85 | 2383.33 | 70.41 |
|  |  |  | 1225243.1 | 922014.9 |  |
